# Influence of Surface Roughness on Strong Light-Matter Interaction of a Quantum Emitter-Metallic Nanoparticle System

**DOI:** 10.1038/s41598-018-25584-5

**Published:** 2018-05-08

**Authors:** Yu-Wei Lu, Ling-Yan Li, Jing-Feng Liu

**Affiliations:** 1 College of Electronic Engineering, South China Agricultural University, Guangzhou, 510642 China; 2 School of Physics, Sun Yat-sen University, Guangzhou, 510275 China

## Abstract

We investigate the quantum optical properties of strong light-matter interaction between a quantum emitter and a metallic nanoparticle beyond idealized structures with a smooth surface. Based on the local coupling strength and macroscopic Green’s function, we derived an exact quantum optics approach to obtain the field enhancement and light-emission spectrum of a quantum emitter. Numerical simulations show that the surface roughness has a greater effect on the near-field than on the far-field, and slightly increases the vacuum Rabi splitting on average. Further, we verified that the near-field enhancement is mainly determined by the surface features of hot-spot area.

## Introduction

Light-matter interactions in micro- and nanostructures have attracted much attention in the fields of nanoscience and nanophotonics over the past two decades^1–7^. Metallic nanoparticles (MNPs) and their composite structures supporting localized surface plasmon resonance (LSPR) at specific wavelengths can greatly enhance local electromagnetic fields well below the diffraction limits^8–13^. The strong field enhancement near MNPs makes them one of the ideal candidates for tailoring light-matter interaction and promising in various applications, such as plasmon-controlled spontaneous emission and fluorescence^14–16^, single-molecule detection^17–19^, generation of single quantized plasmon^20–22^, resonance energy transfer^23–25^ and quantum bit entanglement^26–28^, the optical trapping of bacteria and other biological materials^29^, and nanoscale nonlinear optical conversion^30,31^.

Many studies have focused on realizing strong coupling between a quantum emitter (QE) and nanophotonic environments^7,32–36^. Nanoparticle-on-mirror (NPoM)^7,37,38^ and coupled spheres^39^ plasmonic nanostructures with sub-nanometer separation can show strong plasmon-exciton interaction. However, in practice, all fabricated MNPs suffer from nanoscale size inaccuracies and surface roughness that make the distance of QE and nanostructures uncertain^40–44^. Previous studies have shown the differences between the optical response properties of realistic and idealized MNPs^45–50^. Specifically, the surface roughness has a slight impact on the far-field response like the scattering cross section^41,46,47,49,51–53^, whereas the near-field enhancement strongly depends on surface features^43,48,49,54^. Therefore, it is reasonable to expect that the imperfect shape of MNPs will modify the light-matter interaction, especially when QEs are very close to MNPs. However, to the best of our knowledge, previous studies have only investigated weak coupling or the far-field response, and the role of surface roughness in the strong coupling regime are still missing in both theory and experiment.

**Figure 1:**
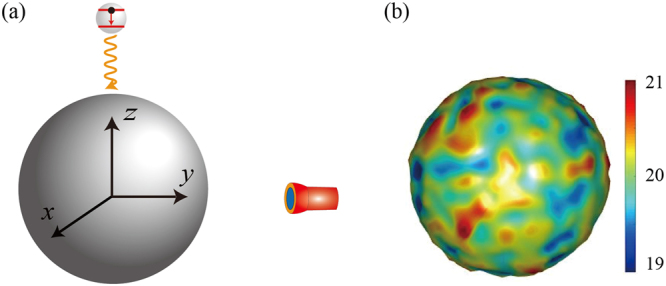
(**a**) Schematic of a 20-nm-radius MNP embedded in vacuum couples with a two-level QE. The QE is located 22 nm above the MNP center along the z-axis. A far-field spectrum detector is placed 1020 nm away from the MNP center. (**b**) Realization of rough MNPs with surface roughness parameters *σ* = 10 nm and Δ*h* = 1 nm.

In this work, we show how surface roughness modifies the strong plasmon-QE interaction. For this purpose, a QE is placed just a few nanometers away from an MNP with rough surface. To capture the essential features of surface roughness, we consider a simple model in which a QE coupled to a 20-nm-radius MNP^55^. For detecting the optical response, a far-field spectrum detector is placed 1000 nm away from the MNP, as shown in Fig. 1(a). The distance between the QE and the center of the idealized MNP is 22 nm, and the dielectric function of the MNP is characterized by the Drude mode ${{\rm{\varepsilon }}}_{m}={{\rm{\varepsilon }}}_{\infty }-{\omega }_{m}^{2}/({\omega }^{2}+i\omega {\gamma }_{m})$. We choose typical parameters for silver with ε_∞_ = 6, *ω*_*m*_ = 7.90 eV and *γ*_*m*_ = 21 meV^39^. The dipole moment of QE is *d* = 24D, and the coupled system is embedded in vacuum. Here, the MNP size and the distance between the MNP and the QE (>1 nm) are in which the Drude model very accurately describes the optical response of a metal^56^. To simulate the surface roughness of realistic MNPs in experiments, we generate Gaussian random fluctuation on the MNP surface that obeys Gaussian distribution with two input parameters: variance of height fluctuation *σ*^2^ and maximum height variation Δ*h* (see Methods section for more details). In this study, we use the parameters *σ* = 10 nm and Δ*h* = 1 nm; however, the mean radius of rough MNP is still 20 nm, which agrees with the radius of an idealized MNP. Figure 1(b) shows one realization of rough MNPs with *σ* = 10 nm and Δ*h* = 1 nm.

## Results

### Theory

To describe quantum light-matter interaction, we derive a general formalism for obtaining the spontaneous emission properties in a fully quantized approach for both emitters and optical field. We consider the interaction of a two-level QE and MNPs; therefore, we adopt a self-consistent procedure of electromagnetic field in absorbing medium based on the dyadic Green function^57–59^. Under this framework, the electric field operator is given by

1 $${\hat{{\bf{E}}}}^{\dagger }({\bf{r}})=i\sqrt{\frac{\hslash }{\pi {\varepsilon }_{0}}}{\int }_{0}^{{\rm{\infty }}}d\omega \frac{{\omega }^{2}}{{c}^{2}}\int {d}^{3}{\bf{r}}^{\prime} \sqrt{{\varepsilon }_{I}({\bf{r}}^{\prime} ,\omega )}{\bf{G}}({\bf{r}},{\bf{r}}^{\prime} ,\omega )\cdot \hat{{\rm{f}}}({\bf{r}}^{\prime} ,\omega )$$

where *ω* is the angular frequency, *c* is the speed of light. *ε*_0_ is the vacuum permittivity, and *ε* and *ε*_*I*_ are respectively the permittivity and its imaginary part of the MNP. ${\hat{{\bf{f}}}}^{\dagger }$ and $\hat{{\bf{f}}}$ are respectively the creation and annihilation operators of the total electric-field represented by a continuum of harmonic oscillators. The dyadic Green’s function **G** (**r**, **r**′, *ω*) in Equation (1) denotes the field with frequency *ω* at **r** responding to a point source located at **r**′. The general definition of **G** (**r**, **r**′, *ω*) is the solution of the wave equation ∇ × ∇ × **G** (**r**, **r**′, *ω*) − (*ω*/*c*)^2^*ε* (**r**, *ω*) **G** (**r**, **r**′, *ω*) = **I***δ* (**r** − **r**′), where **I** is the unit tensor.

By using Equation (1), the system Hamiltonian under the rotating wave approximation can be written as

2 $$H=\int {d}^{3}{\bf{r}}{\int }_{0}^{\infty }\hslash \omega {\hat{{\bf{f}}}}^{\dagger }({\bf{r}},\omega )\hat{{\bf{f}}}({\bf{r}},\omega )d\omega +\hslash {\omega }_{a}|e\rangle \langle e|-({\hat{\sigma }}^{\dagger }{\hat{E}}^{\dagger }({{\bf{r}}}_{a})\cdot {\bf{d}}+h\mathrm{.}c\mathrm{.})$$

where ${\hat{\sigma }}^{\dagger }=|e\rangle \langle g|$ and ${\hat{\sigma }}^{-}=|g\rangle \langle e|$ are the raising and lowering operators of two-level QE, respectively. The QE is located at position **r**_*a*_, with the atom transition frequency *ω*_*a*_ and transition dipole moment **d** = *d****μ***, where ***μ*** is unit orientation vector. |e〉 and |g〉 are the upper and lower states of the QE, respectively.

The temporal evolution of system at time *t* can be obtained by solving the following wave function

3 $$|{\rm{\Psi }}(t)\rangle ={C}_{e}(t)|e\mathrm{,0}\rangle +\int {d}^{3}{\bf{r}}{\int }_{0}^{\infty }d\omega {C}_{g}({\bf{r}},{\boldsymbol{\omega }},t)|g,{\bf{1}}({\bf{r}},{\boldsymbol{\omega }})\rangle $$

where $|e,\,0\rangle$ represents the QE is in excited state and its surrounding is unexcited, and $|g,{\bf{1}}({\bf{r}},{\boldsymbol{\omega }})\rangle$ indicates that the QE has returned to the ground state and the surrounding optical field is excited. Here, we suppose that QE is initially in the upper state and the optical field is in the vacuum, that is, *C*_*e*_(0) = 1 and *C*_*g*_ (**r**, *ω*, 0) = 0. Therefore, we obtain the evolution equations of amplitude *C*_*e*_ (*t*) and *C*_*g*_ (**r**, *ω*, *t*) as

4 $$\begin{array}{rcl}{\dot{C}}_{e}(t) & = & -i{\omega }_{a}{C}_{e}(t)-\sqrt{\frac{1}{\hslash \pi {\varepsilon }_{0}}}{\int }_{0}^{\infty }d\omega \frac{{\omega }^{2}}{{c}^{2}}\int {d}^{3}{\bf{r}}\sqrt{{\varepsilon }_{I}({\bf{r}},\omega )}{\bf{d}}\cdot {\bf{G}}({{\bf{r}}}_{a},{\bf{r}},\omega ){C}_{g}({\bf{r}},\omega ,t)\\ {\dot{C}}_{g}({\bf{r}},\omega ,t) & = & -i\omega {C}_{g}({\bf{r}},\omega ,t)+\sqrt{\frac{1}{\hslash \pi {\varepsilon }_{0}}}\frac{{\omega }^{2}}{{c}^{2}}\sqrt{{\varepsilon }_{I}({\bf{r}},\omega )}{\bf{d}}\cdot {{\bf{G}}}^{\ast }({{\bf{r}}}_{a},{\bf{r}},\omega ){C}_{e}(t)\end{array}$$

By using one-sided Fourier transformation^60,61^, the explicit solution of *C*_*e*_ (*t*) in frequency domain can be expressed as

5 $${C}_{e}(\omega )=\frac{{\rm{\Gamma }}({{\bf{r}}}_{a},\omega \mathrm{)/2}}{{[(\omega -{\omega }_{a})-{\rm{\Delta }}({{\bf{r}}}_{a},\omega )]}^{2}+{[{\rm{\Gamma }}({{\bf{r}}}_{a},\omega \mathrm{)/2]}}^{2}}$$

with

6 $${\rm{\Gamma }}({{\bf{r}}}_{a},\omega )=\frac{2{\omega }^{2}}{\hslash {\varepsilon }_{0}{c}^{2}}{\bf{d}}\cdot {\bf{I}}{\rm{m}}\,[{\bf{G}}({{\bf{r}}}_{a},{{\bf{r}}}_{a},\omega )]\cdot {\bf{d}}$$

and

7 $${\rm{\Delta }}({{\bf{r}}}_{a},\omega )=\frac{1}{2\pi }{\mathscr{P}}{\int }_{0}^{\infty }\frac{{\rm{\Gamma }}({{\bf{r}}}_{a},\omega ^{\prime} )}{\omega -\omega ^{\prime} }d\omega ^{\prime} $$

where ${\mathscr{P}}$ denotes the principal value. *C*_*e*_ (*ω*) is the local spontaneous emission spectrum (LSES) of QE that corresponds to polarization spectrum^55,62^ or dipole spectrum^63^. Γ(**r**_*a*_, *ω*) and Δ(**r**_*a*_, *ω*) are the local coupling strength (LCS)^60,61,64,65^ and level shift^2,55,66,67^, respectively. The LCS is usually expressed in terms of the local density of states (LDOS) which is defined as^68^

8 $${\rm{\rho }}({{\bf{r}}}_{a},\omega )=\frac{6\omega }{\pi {c}^{2}}{\boldsymbol{\mu }}\cdot {\rm{Im}}\,[{\bf{G}}({{\bf{r}}}_{a},{{\bf{r}}}_{a},\omega )]\cdot {\boldsymbol{\mu }}$$

ρ (**r**, *ω*) is also called the projected LDOS because **G**(**r**_*a*_, **r**_*a*_, *ω*) is projected into ***μ*** direction, which is, the orientation of the transition dipole moment of QE^55^. In vacuum, the LDOS is ρ_0_ = *ω*^2^/(*π*^2^*c*^3^). Then, LCS can be rewritten as Γ(**r**_*a*_, *ω*) = *πωd*^2^/(3*ħε*_0_)ρ(**r**_*a*_, *ω*).

LDOS is an essential quantity for describing the emission characteristics of QE from weak to strong coupling regime in micro- and nanostructures^69,70^. Therefore, it is necessary to reveal the influence of surface roughness on LDOS. Moreover, it is widely accepted that the strong coupling is inextricably linked to high field enhancement; therefore, we use the definition of the enhancement of LDOS

9 $${F}_{i}=\frac{{\mu }_{i}\cdot \,{\bf{I}}{\rm{m}}\,[{\bf{G}}({{\bf{r}}}_{a},{{\bf{r}}}_{a},\omega )]\cdot {\mu }_{i}}{{\mu }_{i}\cdot \,{\bf{I}}{\rm{m}}\,[{{\bf{G}}}_{0}({{\bf{r}}}_{a},{{\bf{r}}}_{a},\omega )]\cdot {\mu }_{i}}$$

where ${\bf{I}}{\rm{m}}\,[{{\bf{G}}}_{0}({{\bf{r}}}_{a},{{\bf{r}}}_{a},\omega )]=k\sqrt{{\varepsilon }_{b}}/(6\pi ){\bf{I}}$ is the imaginary part of Green’s tensor in homogeneous medium. Here *ε*_*b*_ is the dielectric constant of homogeneous medium.

The far-field detected spectrum (FFDS) of the emitted light by QE is defined through a double-time integration over the first order quantum correlation function ${\rm{s}}({{\bf{r}}}_{d},\omega )=\int d{t}_{2}\int d{t}_{1}{e}^{-i\omega ({t}_{2}-{t}_{1})}\langle \hat{{\bf{E}}}({{\bf{r}}}_{d},{t}_{2}){\hat{{\bf{E}}}}^{\dagger }({{\bf{r}}}_{d},{t}_{1})\rangle$^55,63^, where $\hat{{\bf{E}}}={({\hat{{\bf{E}}}}^{\dagger })}^{\dagger }$ and **r**_*d*_ is the location of detector. By substituting Equations (1) and (3) into the expression of *s* (**r**_*d*_, *ω*), we can rewrite the FFDS as ${\rm{s}}({{\bf{r}}}_{d},\omega )={|\int dt{e}^{-i\omega t}{\hat{{\bf{E}}}}^{\dagger }(r)|{\rm{\Psi }}(t)\rangle |}^{2}$. Therefore, it is straightforward to obtain the analytical expression of FFDS.

10 $${\rm{s}}({{\bf{r}}}_{d},\omega )={{\mathscr{G}}}_{r}({{\bf{r}}}_{d},{{\bf{r}}}_{a},\omega ){C}_{e}(\omega )$$

where ${{\mathscr{G}}}_{r}({{\bf{r}}}_{d},{{\bf{r}}}_{a},\omega )={|{\omega }^{2}{\bf{G}}({{\bf{r}}}_{d},{{\bf{r}}}_{a},\omega )\cdot {\boldsymbol{\mu }}/({\varepsilon }_{0}{c}^{2})|}^{2}$ is the propagation function from QE to detector. The propagation function describes the essential role of light propagation in FFDS through the Green’s propagator **G** (**r**_*d*_, **r**_*a*_, *ω*) for two space-point. Equation (10) clearly shows that FFDS contains information about both the local dynamics and the propagation process.

For investigating the influence of surface roughness on Green’s propagator **G** (**r**_*d*_, **r**_*a*_, *ω*), we define a factor, in a manner similar to Equation (9), called far-field enhancement (FFE).

11 $${F}_{i}^{d}=\frac{|{\bf{G}}({{\bf{r}}}_{d},{{\bf{r}}}_{a},\omega )\cdot {\boldsymbol{\mu }}|}{{\mu }_{i}\,\cdot \,{\bf{I}}{\rm{m}}\,[{{\bf{G}}}_{0}({{\bf{r}}}_{a},{{\bf{r}}}_{a},\omega )]\cdot {\mu }_{i}}$$

At the end of this section, we further explain our theoretical method. First, without making Markovian approximation, the LSES and FFDS are valid in both the weak and the strong coupling regime. Second, we can obtain the reversible dynamics of QE in the strong coupling regime^71–73^ through the Fourier transformation of LSES *C*_*e*_ (*ω*). Finally, our method can also be extended to multiple QEs system to find out the underlying cooperative effect under strong light-matter interaction.

Based on the above theoretical method, in the following section, we study the differences in optical response between MNPs with smooth surface and rough surface. For evaluating the LCS and propagation function in Equation (10), we choose the MNPBEM toolbox^74–76^ based on the boundary element method (BEM) as the numerical simulation platform. The BEM approach only requires discretizing the MNP surface, making it extremely suitable and efficient for plasmonics studies^47,49^. Hereafter, we use a dipole emitter to represent the two-level QE.

### Near- and Far-Field Enhancement

**Figure 2:**
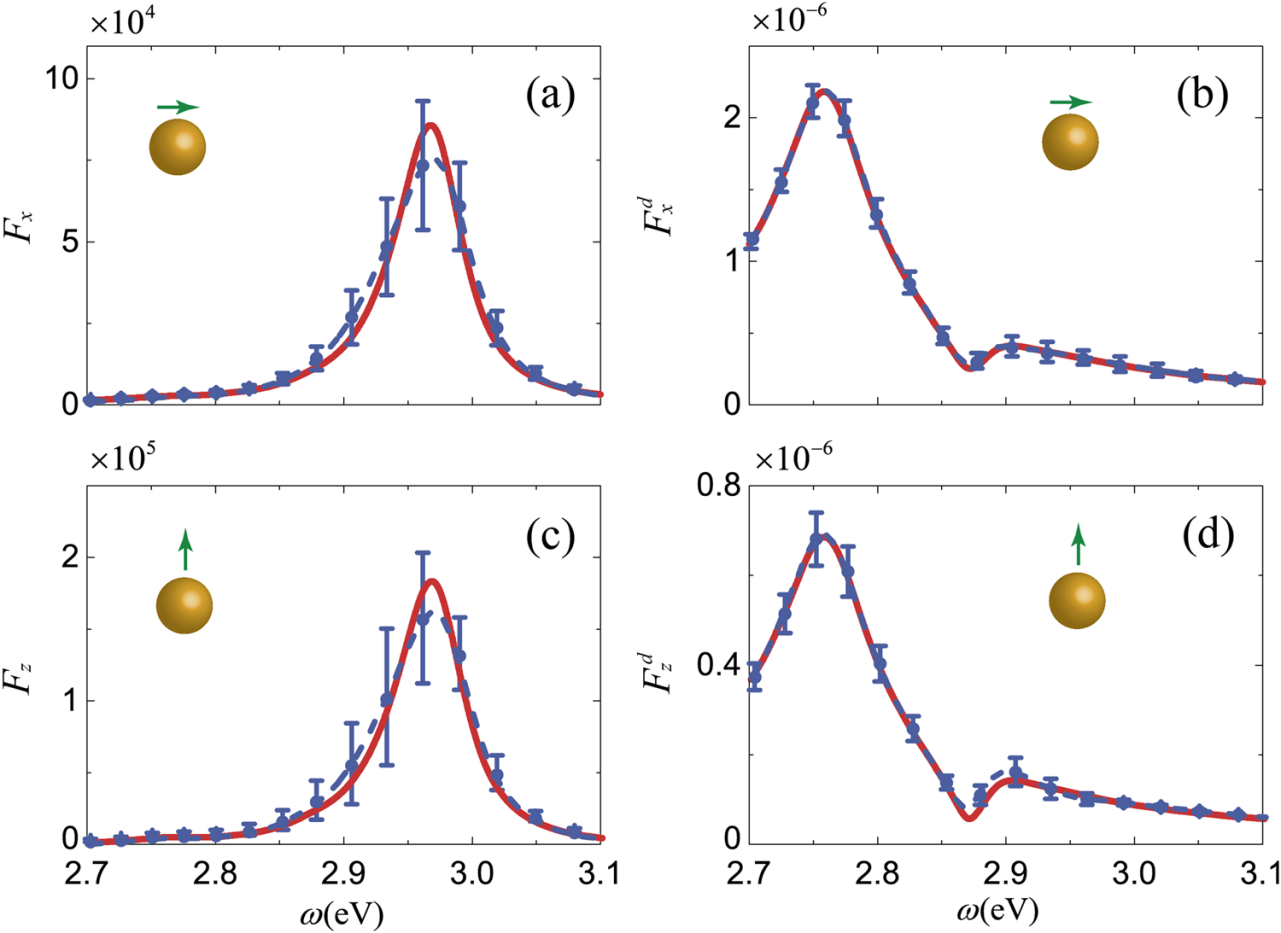
(**a**) *F*_*x*_ averaged over 100 random realizations of rough MNPs (blue dashed line with error bar). *F*_*x*_ of idealized MNP is shown for comparison (red solid line). Insert: green arrow indicates the dipole orientation. (**b**) Far-field enhancement ${F}_{x}^{d}$ averaged over 100 random realizations of rough MNPs (blue dashed line with error bar). The result of idealized MNP is shown for comparison (red solid line). In (**a**) and (**b**) we use an x-oriented dipole. (**c**) and (**d**) are the same as (**a**) and (**b**) but for a z-oriented dipole.

Figure 2(a) and (c) show the enhancement of LDOS averaged over 100 rough MNPs for x- and z-oriented dipoles, respectively. The x- and z-oriented dipoles are parallel and perpendicular to the MNP, respectively, as shown in the inset in Fig. 2. The vertical error bar in figures denotes the standard error of the mean. The error bar in Fig. 2(c) is longer than that in Fig. 2(a), indicating that LDOS is more sensitive to surface features when the dipole is perpendicular to the MNP. The same conclusion can also be drawn from far-field enhancement, as indicated by the longer error bar in Fig. 2(d) than in Fig. 2(b). A comparison of the standard error of averaged LDOS and that of far-field enhancement clearly shows that the surface roughness produces a comparatively small effect in the far-field and a dramatic one in the near-field. Furthermore, we find that the LDOS of rough MNPs reduces by ~10% and ~12% on average for x- and z-oriented dipoles compared to idealized MNPs, respectively. Because the surface roughness can shift the resonant peak of LDOS, the averaged LDOS of rough MNPs is reduced and broadened slightly compared to that of idealized MNPs. However, the peak frequency shows no visible change between idealized and rough MNPs in terms of both LDOS and far-field enhancement.

### Local Surface Features Analysis

The above results indicate that the height fluctuation of the surface greatly impacts the near-field enhancement. However, it is difficult to directly determine what type of rough surface can increase the strength of the light-matter interaction. We know that a tip on surface should enhance LDOS owing to the lightning rod effect, whereas the magnitude of enhancement and existence of other effects remain unknown.

**Figure 3:**
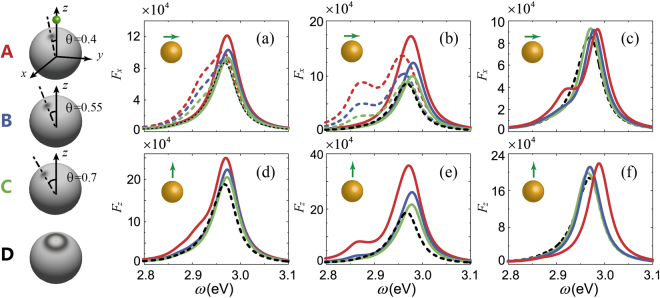
*F*_*i*_ of idealized MNP with a bump. Left: models of idealized MNP with a bump located along the x-direction for *θ* = 0.4 rad (**A**), *θ* = 0.55 rad (**B**) and *θ* = 0.7 rad (**C**). Green dot in (**A**) represents the dipole. (**D**) is the modal of an idealized MNP with ring bump. Right: (a) *F*_*x*_ of MNP with a 0.5 nm-high-bump along the x-direction (dashed line) and y-direction (solid line) using an x-oriented dipole for *θ* = 0.4 rad (red line), *θ* = 0.55 rad (blue line) and *θ* = 0.7 rad (green line). (b) is the same as (a) except that the bump has 1.0-nm height. (c) *F*_*x*_ of MNP with a ring bump with 0.5-nm height using an x-oriented dipole for *θ* = 0.4 rad (red line), *θ* = 0.55 rad (blue line) and *θ* = 0.7 rad (green line). (d–f) shows results for a z-oriented dipole with the other parameters being the same as those in (a–c). *F*_*i*_ of idealized MNPs without a bump is shown for comparison (black dashed line).

In this section, we investigate the optical properties of the MNP with a bump. The bump is placed on the MNP surface at various positions and different heights to change the bump–dipole distance. Considering the dipole orientation and symmetry of the sphere, the bump is sited along both x- and y-directions when using an x-oriented dipole but only along the y-direction when using a z-oriented dipole. Figure 3((A–C), left-hand side) shows a schematic diagram of a bump sited along the x-direction at various positions. Figure 2(b) and (d) indicate that the surface roughness has almost no effect on the far-field, therefore, we focus on the effect of the bump on near-field enhancement.

Figure 3(a,b) shows LDOS for MNPs with a small bump for an x-oriented dipole, and Fig. 3(d,e) shows that for a z-oriented dipole. The bump height is 0.5 nm in Fig. 3(a) and (d) and 1.0 nm in Fig. 3(b) and (c). The numerical results clearly show that all LDOSs with bumps are larger than that of the idealized MNP (black dashed line in figures). For the same bump height and direction, the shorter bump–dipole distance, the larger LDOS; for a fixed bump position, LDOS for a 1-nm high bump is larger than that for a 0.5 nm high bump. Therefore, the numerical results confirm that the bump enhances the LDOS, and it shows larger enhancement with shorter bump–dipole distance. In our configuration, the largest the enhancement of LDOS is produced by a bump with 1 nm height and *θ* = 0.4 rad, whose LDOS is nearly two times that of an idealized MNP. When the bump is far from the dipole, for example, with *θ* = 0.7 rad (green line in Fig. 3), LDOS of MNPs with a bump converges with that of idealized MNPs. We also note that for a bump with the same height and position, the LDOS for a z-oriented dipole is slightly larger than that for an x-oriented dipole. This agrees with the result in the previous section that the impact of surface roughness is greater when the dipole is perpendicular to MNP.

**Figure 4:**
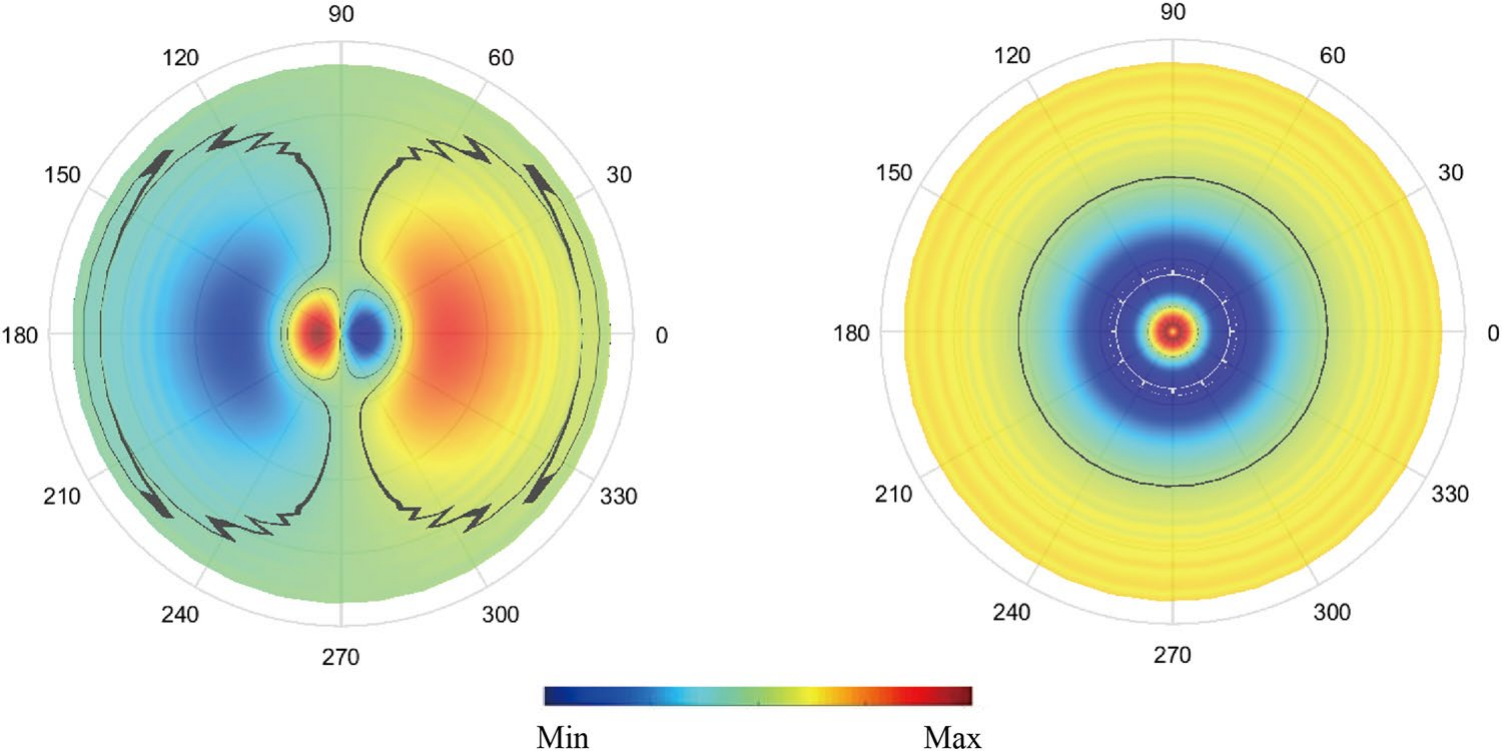
Electric field amplitude on hot-spot area of idealized MNP for an x-oriented dipole (left) and for a z-oriented dipole (right) with transition frequency *ω*_*a*_ = 2.963 eV; this coincides with the LSPR resonant frequency. The black line indicates 10% of the maximum electric field intensity.

Figure 3(a) and (b) show the peak of LDOS red shifts when a bump is located along the x-direction for an x-oriented dipole; the red-shifting is larger if the bump is closer to the dipole. However, the peak is blue-shifted for a bump located along the y-direction. The blue-shifting changes nonmonotonously with *θ* and has a maximum of ~0.013 eV at *θ* = 0.55 rad (blue line in Fig. 3). Similar behavior is also found when using a z-oriented dipole, as shown in Fig. 3(d) and (e). In this case, the maximum blue-shifting is ~0.011 eV. In addition, the bump direction is nonsignificant because the electric field distribution is circularly symmetric (right panel of Fig. 4).

It is worth to point out that a bump on the surface broadens LDOS owing to the appearance of a new plasmon mode at lower energy, and the secondary peak shows larger red-shifting with increasing bump height. This can be seen from a comparison of the red line in Fig. 3(d) with that in Fig. 3(e). For a 1-nm-high bump located along the x-direction with an x-oriented dipole, the secondary peak combined with the main peak of LDOS produces a broad range of enhancement of up to nearly 10^5^ (red dashed line in Fig. 3(b)). This feature may enable further exploring the possibility of expanding and controlling the range of enhancement of LDOS. This new plasmonic mode appears because of the bump breaking the surface symmetry. To support this claim, we recover the surface symmetry by replacing the single bump by a ring bump (D, left-hand side of Fig. 3). The LDOS of an MNP with a ring bump shows no obvious broadening compared to that of an idealized MNP for various *θ*, as shown in Fig. 3(c) and (f).

Briefly, our calculations show that a bump close to the dipole can increase the LDOS magnitude, shift the resonant frequency slightly, and broaden the LDOS.

The results shown in Figs 2 and 3 indicates that a bump generally takes effect when it is close enough to the dipole. Therefore, the LDOS of rough MNPs may primarily be determined by the surface features of the hot-spot area because of the short distance to the dipole. Here, the hot-spot area is defined as the surface in the range of $0\ll \theta \ll \pi \mathrm{/8}$, where *θ* is the angle of zenith in spherical coordinates. We choose this range to guarantee that the localized surface plasmon energy is predominately located in this region, as shown in Fig. 4. The origin of the spherical coordinates is the MNP center, and the dipole location is (22 nm, 0, 0).

**Figure 5:**
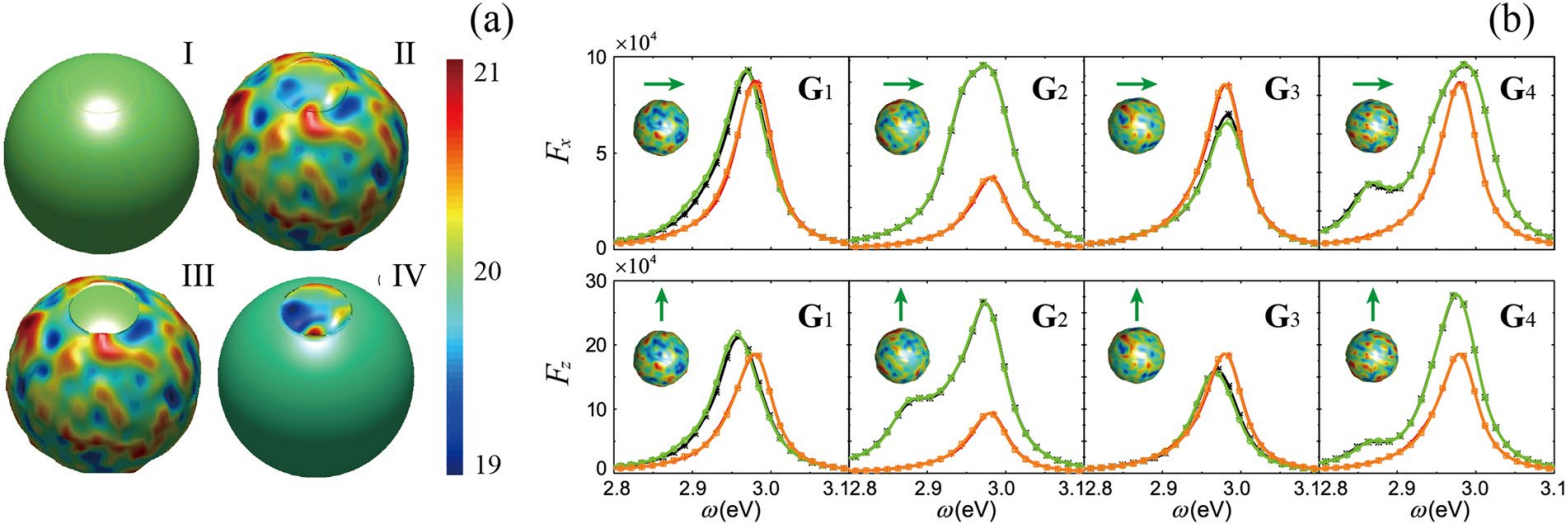
(**a**) Schematic diagram of a simulation group. Every simulation group contains four types of MNP: smooth MNP (I), rough MNP (II), smooth-rough MNP (III), and rough-smooth MNP (IV), as discussed in text. MNPs within a group use identical realization of the rough surface. (**b**) LDOS of four simulation groups *G*_1_ − *G*_4_ for x-oriented dipole (upper panels) and z-oriented dipole (lower panels). The green line with crosses and black line with stars indicate the results of rough and rough-smooth MNPs, respectively; the orange line with crosses corresponds to smooth MNPs and the red line with stars corresponds to smooth-rough MNPs. Inset: dipole orientation and rough MNP (model (II) in panel (a)) in each group.

To verify the above hypothesis, we need to compare the difference in LDOS of two MNPs whose hot-spot areas are identical whereas other surface areas are significantly different. Therefore, we create a partially smooth-rough MNP (*smooth-rough MNP*, model (III) in Fig. 5(a)) by smoothing the rough surface of the hot-spot area for comparison with an idealized MNP (*smooth* MNP, model (I)). For contrast, we also generate an idealized MNP with a rough hot-spot area (*rough-smooth* MNP, model (IV)) and compare it to a completely *rough* MNP (model (II)). Then, we use these four types of MNPs to form a simulation group; in each group, we use identical realization of the rough surface. Additionally, to avoid the discontinuity of the junction of two areas, we use the interpolation method. Figure 5(a) shows an example of a simulation group.

We generate four simulation groups, *G*_1_ − *G*_4_, to perform numerical simulations. Figure 5(b) shows the corresponding results. According to the hypothesis, the LDOS of smooth MNP and smooth-rough MNP and that of rough MNP and rough-smooth MNP should share similar features, respectively. The numerical results show the predicted phenomenon: *F*_*i*_ of MNPs with the same surface features as the hot-spot area within a group only shows a minor difference. However, a new plasmonic mode may only appear for a completely rough MNP but disappear for a partially rough MNP, like the rough-smooth MNP. However, in most cases, the surface features of hot-spot area are crucial.

### Near- and Far-Field Spectra

In this section, we study the influence of surface roughness on the emission spectra. The LSES and FFDS for rough MNPs are calculated with a specific transition frequency *ω*_*a*_ and averaged. We only focus on the influence of surface roughness on the peak frequency, as indicated by the triangle with the horizontal error bar in the emission spectra.

**Figure 6:**
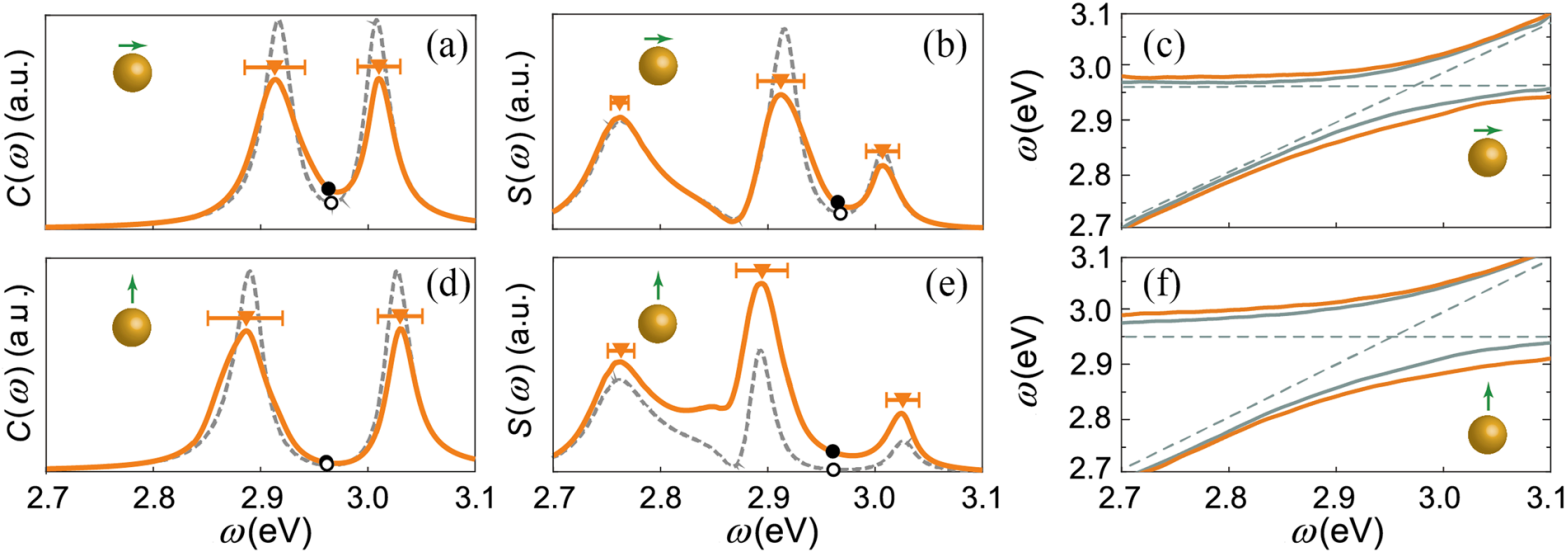
(**a**) LSES for idealized MNP (gray dashed line) and for rough MNPs on average (orange solid line). (**b**) FFDS for idealized MNP (gray dashed line) and for rough MNPs on average (orange solid line). (**c**) LSES spectral peaks as a function of frequency averaged over 100 random realizations of rough MNPs (orange solid line). LSES spectral peaks for idealized MNP are also shown (gray solid line). The gray dashed line represents the uncoupled transition frequencies. (**a–c**) x-oriented dipole. (**d**–**f**) z-oriented dipole. The upside-down triangle in the error bar indicates the peak frequency, and the error bar indicates the standard deviation of the mean. Transition frequencies are indicated by black dots and hollow dots on the curves for MNPs with a bump and idealized MNP, respectively.

Figure 6(a) and (d) show the averaged LSES of rough MNPs for x- and z-oriented dipoles, respectively. For an idealized MNP, of the splitting of LSES are ~92 meV for an x-oriented dipole and ~138 meV for a z-oriented dipole. Numerical simulation results show that the averaged splitting of rough MNPs is larger in both cases, being ~95 meV for an x-oriented dipole and ~143 meV for a z-oriented dipole. The splitting enlarges because both peaks in the averaged LSES shift outward: the peak at lower energy is red-shifted whereas that at higher energy is blue-shifted. We note that the peaks in the LSES also remain in the FFDS; however, light emitted to the far-field adds an additional peak at ~2.77 eV, as shown in Fig. 6(b) and (e). This additional peak is caused by the propagation function ${{\mathscr{G}}}_{r}({{\bf{r}}}_{d},{{\rm{r}}}_{a},\omega )$, whose features are seen in Fig. 2(b) and (d). The error bars of the LSES and FFDS clearly indicate that the peaks in the FFDS are less affected by the surface roughness than the peaks in the LSES, confirming the former conclusion that the surface roughness has less effect on the far-field. Moreover, the deviations of the frequency of the peaks in FFDS between idealized and rough MNPs are too small to observe.

The anticrossing behavior of LSES is the footprint of strong coupling. To examine the anticrossing behavior of LSES, we locate the maximum of peaks in the LSES with various transition frequencies, and we show the results in Fig. 6(c) and (f). We observe a small increase in the vacuum Rabi splitting after introducing the surface roughness for both dipole orientations; however, the increase for the z-oriented dipole is larger. It should be noted that in the anticrossing curves of rough MNPs, greater deviation from idealized MNP emerges at band energy of ~2.96 eV that approaches the LSPR resonant frequency. This agrees with the averaged LDOS for rough MNPs shown in Fig. 2, in which the standard error and deviation are both relatively large near the resonant frequency.

**Figure 7:**
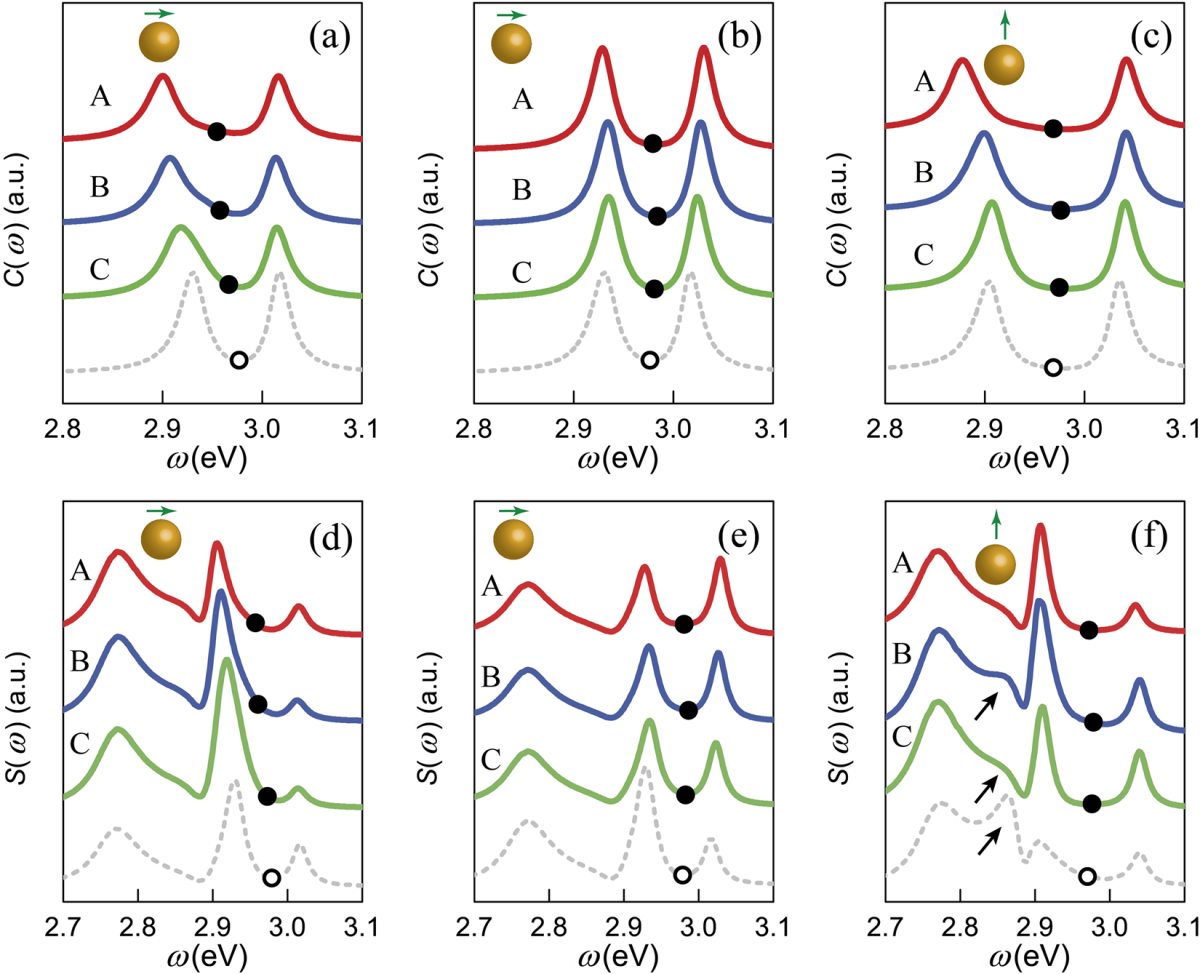
LSES of idealized MNP with a 0.5-nm-high bump located along the x-direction (**a**) and y-direction (**b**) for x-oriented dipole and along the y-direction for z-oriented dipole (**c**). Curves A, B, and C correspond to models A, B, and C in Fig. 3, respectively, whose *θ* values are 0.4 rad (red line), 0.55 rad (blue line), and 0.7 rad (green line). (**d**–**f**) are the corresponding FFDS for models (**a–c**). Transition frequencies are indicated by black dots and hollow dots on the curves for MNPs with a bump and idealized MNP, respectively.

Finally, we investigate how a single bump affects the features of LSES and FFDS. The models are the same as those shown in Fig. 3 and Fig. 7 shows the results of the MNP with a 0.5-nm-high bump. The bump enlarges splitting in both the LSES and the FFDS. Therefore, the existence of a bump is beneficial for achieving strong coupling, especially for a z-oriented dipole and small *θ*. For an x-oriented dipole, when the bump is located along the x-direction, the splitting in the LSPS and FFDS is larger than the bump located along the y-direction while other conditions remain the same. It is generally more difficult to observe the Rabi splitting in FFDS because of the broadening and asymmetry of the peaks. However, we note that when the bump is located along the y-direction, the splitting is more symmetric compared to that in an idealized MNP, as shown in Fig. 7(e). This situation occurs because the bump enhances the peak at higher energy, which is difficult to observe for an idealized MNP. Furthermore, the bump can narrow the peaks and make the splitting in FFDS easy to observe with a z-oriented dipole. This can be seen by comparing the gray dashed line and solid lines in Fig. 7(f). In addition, Fig. 7 also shows that the lower-energy peak of splitting shows larger shifting than a high-energy peak as *θ* decreases from 0.7 rad to 0.4 rad. This is because the lower-energy peak is ~2.9 eV and is close to the peak of LDOS, where the variance is larger; therefore, it is susceptible to the surface roughness. By contrast, the peak located at ~2.77 eV in the FFDS shows negligible change. It should be noted that the FFDS for an idealized MNP with a z-oriented dipole has a peak near 2.85 eV, as indicated by the black arrows in Fig. 7(f). This peak is reduced by the bump even with *θ* = 0.7 rad; it is a feature of the bump that can be observed in the FFDS.

## Discussion

Our comparative study of the field enhancement and emission spectra between idealized MNP and rough MNP clearly demonstrates the influence of surface roughness on strong light-matter interaction. In determining the far-field response, an idealized model is adequate even the QE is close to MNP. By contrast, the difference becomes substantial referred to near-field enhancement. The magnitude of the field and its characteristics, such as resonant frequency and plasmonic mode, may change dramatically. In particular, three important features are noticeable. First, the surface roughness can slightly increase the vacuum Rabi splitting on average. Second, the near-field enhancement is strongly dependent on the local surface features of the hot-spot area; however, the random fluctuation of the surface may create a new plasmonic mode and make the LDOS and LSES unpredictable. Finally, the FFDS originates from LSES but is weighted by the propagation function ${{\mathscr{G}}}_{r}({{\bf{r}}}_{d},{{\bf{r}}}_{a},\omega )$. The splitting in LSES is in the region of the high-order LSPR mode as it dominates the dynamics of QE that strongly couples with MNP; however, the peaks of the propagation function are predominately located at the dipole LSPR. This mismatch reduces the impact of surface roughness on the far-field and makes the FFDS steady.

In summary, we have studied the influence of surface roughness on near- and far-field responses by simulating plenty of rough MNPs coupled with QE. Both near- and far-field enhancement of QE can be investigated using the BEM method. The LDOS may be greatly influenced by the surface roughness; however, on average, the LDOS and anticrossing are robust to the surface roughness of MNPs. Though the surface roughness of MNPs has obvious effect on LSES, its effect on FFDS is negligible. The results of the current study are obtained at a QE–MNP distance of 2 nm; however, there is no significant difference and limitations in applying it to a ultranarrow gap in composite systems like dimers and NPoM, except for the consideration of nonlocal damping^77–83^. Our research is of practical importance, and it may be inspiring and helpful in designing experiments for strong plasmon-QE interactions.

## Methods

### Generation of Gaussian Random Rough Surface

We generate the Gaussian random fluctuation by attaching the Gaussian stochastic phase factor to the Fourier coefficients in the spectrum density, which is defined as

12 $$H({k}_{x},{k}_{y})={\sigma }^{2}{e}^{i{\rm{\Delta }}\varphi }(\frac{{l}_{x}}{2\sqrt{\pi }}{e}^{-\frac{{k}_{x}^{2}{l}_{x}^{2}}{4{\sigma }^{2}}})(\frac{{l}_{y}}{2\sqrt{\pi }}{e}^{-\frac{{k}_{y}^{2}{l}_{y}^{2}}{4{\sigma }^{2}}})$$

where *σ*^2^ is the variance of the height fluctuation, *l*_*x*_ and *l*_*y*_ are the correlation length along x- and y-directions, respectively, and Δ*ϕ* is the Gaussian stochastic variable. Then, the rough surface whose height obeys the Gaussian distribution can be obtained by the inverse Fourier transformation

13 $$h(x,y)={\rm{\Delta }}h\cdot {\rm{Re}}\,[{ {\mathcal F} }^{-1}[H({k}_{x},{k}_{y})]]$$

where ${ {\mathcal F} }^{-1}$ denotes the inverse Fourier transformation, Δ*h* is the maximum height variation of the surface roughness. Finally, *h*(*x*, *y*) is projected on the surface of an idealized MNP to obtain a rough MNP.

### Data availability

The datasets generated and analysed during the current study are available from the corresponding author on reasonable request.
